# The Role of Natural Antioxidant Products That Optimize Redox Status in the Prevention and Management of Type 2 Diabetes

**DOI:** 10.3390/antiox12061139

**Published:** 2023-05-23

**Authors:** Dawn S. Tuell, Evan A. Los, George A. Ford, William L. Stone

**Affiliations:** Department of Pediatrics, Quillen College of Medicine, Johnson City, TN 37614, USA

**Keywords:** antioxidants, oxidative stress, reactive oxygen species, type 2 diabetes, pediatrics, redox, glycemic control, exercise, lifestyle, mitochondria

## Abstract

The worldwide prevalence of type 2 diabetes (T2D) and prediabetes is rapidly increasing, particularly in children, adolescents, and young adults. Oxidative stress (OxS) has emerged as a likely initiating factor in T2D. Natural antioxidant products may act to slow or prevent T2D by multiple mechanisms, i.e., (1) reducing mitochondrial oxidative stress, (2) preventing the damaging effects of lipid peroxidation, and (3) acting as essential cofactors for antioxidant enzymes. Natural antioxidant products should also be evaluated in the context of the complex physiological processes that modulate T2D-OxS such as glycemic control, postprandial OxS, the polyol pathway, high-calorie, high-fat diets, exercise, and sleep. Minimizing processes that induce chronic damaging OxS and maximizing the intake of natural antioxidant products may provide a means of preventing or slowing T2D progression. This “optimal redox” (OptRedox) approach also provides a framework in which to discuss the potential benefits of natural antioxidant products such as vitamin E, vitamin C, beta-carotene, selenium, and manganese. Although there is a consensus that early effective intervention is critical for preventing or reversing T2D progression, most research has focused on adults. It is critical, therefore, that future research include pediatric populations.

## 1. Introduction

The worldwide prevalence of diabetes mellitus (DM) over the last three decades has at least tripled, making this disease a critical public health and economic issue for most nations [1,2]. About 90–95% of those with DM have type 2 DM (T2D) which is rapidly increasing in children, adolescents, and young adults along with an increasing rate of prediabetes, a major risk factor for T2D [3]. About one in three USA adults have prediabetes, which is undiagnosed in eight out of ten people [4,5]. The total USA cost of DM in 2017 was USD 327 billion with each diagnosed individual incurring about USD 16,000 in annual medical expenses [4].

While the US Prevention Services Task Force currently recommends screening adults for prediabetes and T2D, it suggests that current evidence is insufficient to assess the balance of benefits and harms of screening for children or adolescents [5]. Of the 37.1 million USA adolescents and adults with DM, it is estimated that about one in five is “undiagnosed” [6]. While individual lifestyle alterations for T2D and prediabetes are generally considered the treatments of choice, this option can be problematic for those without a diagnosis. Moreover, children and adolescents with T2D show a high dropout from the medical care system suggesting the need for some “remodeling of current healthcare practices” [7]. 

As detailed below, considerable evidence supports the role of damaging oxidative stress (OxS) as a key factor in both the initiation and progression of T2D. This review will explore the hypothesis that minimizing physiological factors that induce damaging OxS and maximizing natural antioxidant products (i.e., the OptRedox strategy) may provide a means of preventing or slowing T2D progression. Both adults and the pediatric population could potentially benefit from this OptRedox strategy. Since early intervention is critical, this strategy would be optimally effective if implemented as a public health policy in the pediatric population. School-based T2D interventions are a logical focal point for exploring future OptRedox strategy implementation and research [8]. 

Considering the large number of individuals that have undiagnosed prediabetes or T2D, a “blanket” implementation of OptRedox must be justified by evidence-based medicine. Moreover, some individuals are likely at high risk for OxS-driven T2D, and a systems medicine approach with a complementary emphasis on redoxomics might identify such individuals and predict if they could be responders to OptRedox or other medical treatments [9]. Before detailing the natural OptRedox products for potentially combating T2D, we will first summarize the dynamic involvement of OxS in T2D pathogenesis.

## 2. The Role of Oxidative Stress and T2D Pathogenesis

**Figure 1:**
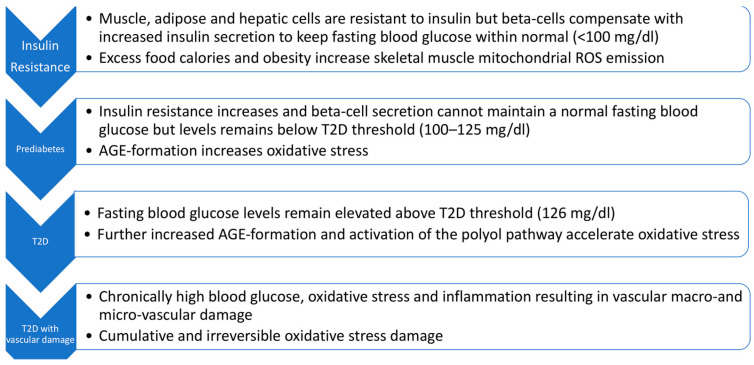
The four stages of type 2 diabetes (T2D) progression and oxidative stress. The roles of reactive oxygen species (ROS) and advanced glycation end products (AGEs) in T2D progression are explained in the text below.

As shown in Figure 1, there are four stages to T2D progression, i.e., insulin resistance, prediabetes, T2D, and T2D with vascular damage [10,11]. As indicated, fasting blood glucose levels (and/or HbA1c) levels are the main criteria for distinguishing these stages. T2D pathogenesis is multifactorial with very complex alterations in carbohydrate, lipid, and protein metabolism [12]. A comprehensive review of T2D pathogenesis is beyond the scope of this review. Viewing T2D as a continuum (as in Figure 1) is, however, useful for promoting interventions at the earliest stage. Fortunately, this is also where the OptRedox strategy may be particularly effective.

## 3. Insulin Resistance in Skeletal Muscle Is Considered the Initiating Defect Leading to T2D

Considerable evidence supports the view that damaging OxS plays a central role in both initiating and accelerating the progression of T2D [9,13,14]. We will focus on the role of OxS in promoting insulin resistance and prediabetes. Insulin resistance in skeletal muscle is considered the initiating, or primary, defect leading to T2D [15]. GLUT4 is the primary glucose transporter for skeletal muscle. Under normal circumstances, insulin binding to the insulin receptor on skeletal muscle cells activates AKT (serine/threonine-specific protein kinases) phosphorylation, eventually resulting in the translocation of GLUT4 to the plasma membrane, thereby promoting glucose transport from plasma into skeletal muscle cells. Skeletal muscle insulin resistance occurs when the normal amount of insulin is inadequate for promoting the expected uptake of glucose. Over time, insulin resistance may result in prediabetes with an increased level of blood glucose. Skeletal muscle accounts for about 80% of insulin-stimulated glucose uptake [15]. Decreased insulin-stimulated translocation of GLUT4 to the skeletal muscle surface, and/or decreased expression of GLUT4, are well-accepted causes of insulin resistance [16,17,18].

## 4. Skeletal Muscle Mitochondrial Hydrogen Peroxide (H_2_O_2_) Emission Results in Insulin Resistance

The pioneering work of Anderson et al. [19] in normal rodents and humans shows that excess dietary calories promote skeletal muscle mitochondrial hydrogen peroxide (H_2_O_2_) emission resulting in transient insulin resistance. While excess dietary carbohydrates and fat both promote this effect, it is markedly more pronounced with a high-fat diet. In obese but otherwise healthy human subjects, skeletal muscle mitochondrial H_2_O_2_ emission was twice the level observed in lean human subjects [19]. 

**Figure 2:**
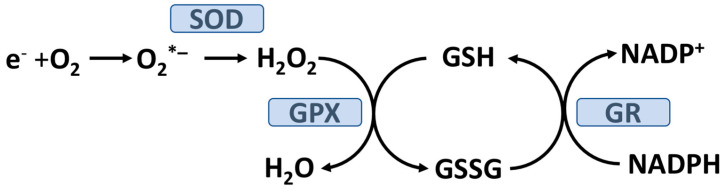
Glutathione peroxidase decomposition of hydrogen peroxide. Mitochondrial electrons from the respiratory chain are donated to oxygen forming the superoxide radical (O_2_*^−^). Superoxide dismutase (SOD) generates hydrogen peroxide (H_2_O_2_) from O_2_*^−^. In the mitochondrial matrix, the only isoform of SOD present is manganese SOD (see text in Section 12). H_2_O_2_ (or lipid hydroperoxide) is reduced to H_2_O (or lipid alcohols) by glutathione peroxidase (GPX) utilizing reduced glutathione (GSH) and generating oxidized glutathione (GSSG). GSSG is recycled back to GSH by glutathione reductase (GR) with the consumption of NADPH. Mitochondrial GPX is a selenium (Se)-requiring enzyme.

A synthetic mitochondrial-specific antioxidant, SS31, was able to block skeletal muscle mitochondrial H_2_O_2_ emission potential as well as the subsequent development of insulin resistance [19]. Mechanistically, a high-fat diet prevented the AKT-mediated translocation of GLUT4 to the skeletal muscle surface as detailed above (other mechanisms could also be involved) [19]. Increased mitochondrial H_2_O_2_ emission was accompanied by a reduction in the skeletal muscle ratio of reduced glutathione (GSH) to oxidized glutathione (GSSG) which would be expected by the glutathione peroxidase (GPX) reduction of H_2_O_2_ to H_2_O (see Figure 2). 

Collectively, the results of Anderson et al. suggest that excess calories, particularly excess fat calories, can result in transitory insulin resistance in normal humans. More recent in vitro and in vivo work by Fazakerley et al. [20] indicates that specifically inducing mitochondrial production of O_2_^−^/H_2_O_2_, by use of paraquat, will also induce insulin resistance in adipocytes and muscle cells by blocking insulin-stimulated GLUT4 translocation to the plasma membrane. Moreover, Fazakerley et al. present evidence suggesting that mitochondrial OxS, in the absence of cytosolic OxS, is sufficient to cause insulin resistance [20]. Natural antioxidants that can inhibit mitochondria-induced insulin resistance are, therefore, high-priority candidates for OptRedox since they have the potential to slow or prevent a key T2D-initiating event. This hypothesis will be explored further below.

## 5. Avoiding High-Fat, High-Calorie Meals Could Be an Effective OptRedox Strategy

The Anderson et al. research strongly suggests that avoiding high-fat, high-calorie meals (and the attendant OxS) could be an effective OptRedox strategy for inhibiting early T2D initiation [19]. Encouragingly, there is evidence showing that a low-calorie diet combined with long-term weight-loss support has the potential to reverse T2D in adults [21,22]. The potential of low-calorie diets in slowing or preventing T2D progression in children has not been reported, underlining the critical need for research in this population. The United States Department of Agriculture (USDA)-National School Lunch Program (NSLP) serves over 30 million children and would be an ideal agency to explore research into the option of providing diets optimized to prevent T2D initiation and progression. Such an intervention would need a careful research design and the voluntary participation of children and their parents/guardians.

## 6. Hyperglycemia, Oxidative Stress, and T2D Progression

In susceptible individuals, skeletal muscle insulin resistance can be a contributing factor to increased postprandial glycemia (PPG) [23,24]. Postprandial glucose can eventually exceed 140 mg/dL (2 h after a carbohydrate-containing meal) and become postprandial hyperglycemia (PPH), which is one of the earliest abnormalities associated with T2D [23].

High levels of PPG are problematic for multiple reasons. PPG, 2 h after lunch, is a better predictor of cardiovascular events and all-cause mortality in T2D than fasting blood glucose levels [25,26]. In an excellent and extensive review, Sottero et al. [24] concluded that chronic postprandial OxS resulting from PPH is a key factor driving T2D progression. The mechanisms connecting hyperglycemia and OxS are detailed below. 

## 7. Hyperglycemia, AGE Formation, the Polyol Pathway, and Oxidative Stress

Glucose can covalently react with lysine, arginine, and N-terminal residues on proteins to form glycation adducts which can further react to form advanced glycation end products (AGEs) [27]. Fasting hyperglycemia or PPH increases AGE formation in plasma proteins as well as proteins on the outer plasma-membrane surface of most cells [28,29,30]. Covalent modification of proteins by glucose can alter their structure and, potentially, their functions. For cells relying on insulin-independent GLUT transporters (e.g., GLUT1) intracellular glucose concentration will increase in response to hyperglycemia, thereby promoting AGE-modification of cytosolic proteins. Both red blood cells and vascular endothelial cells rely on GLUT1, and glucose in these cell types will equilibrate with plasma glucose. Under PPH or fasting hyperglycemia, cells primarily relying on GLUT1 will utilize the polyol pathway and thereby lower intracellular glucose by converting it to fructose. The polyol pathway consumes NADPH which is needed to keep GSH in the reduced form (see Figure 2) required by the GPX system to detoxify H_2_O_2_ (and lipid hydroperoxides). Under hyperglycemic conditions, it has been estimated that about 30% of blood glucose goes through the polyol pathway [31]. 

**Figure 3:**
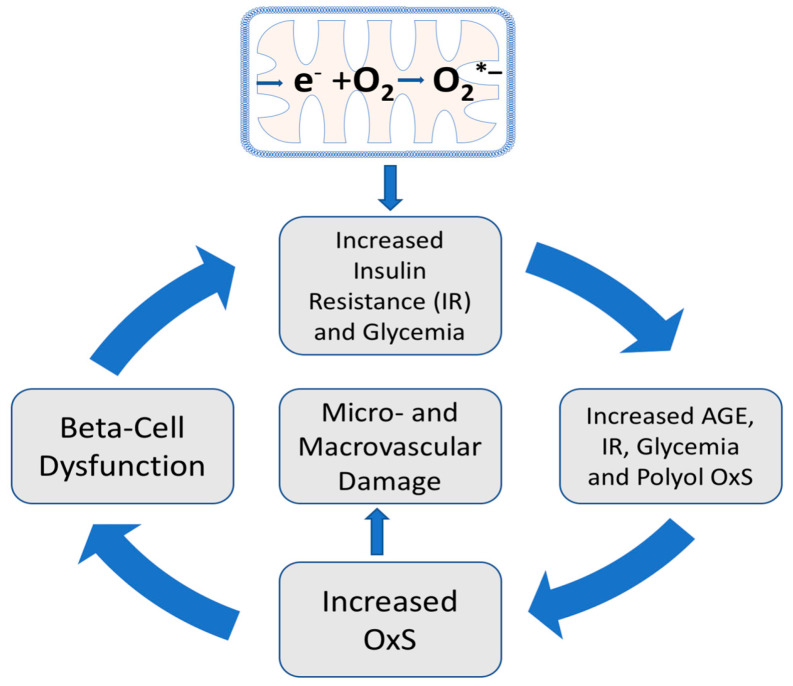
Amplifying oxidative stress (OxS) feedback loops and T2D initiation and progression. Mitochondrial generation of O_2_*^−^ (top) and the subsequent production of H_2_O_2_ (see Figure 2) are proposed initiating factors for T2D and give rise to transient insulin resistance (IR) and increased glycemia. Chronic increases in glycemia promote increased AGE formation which can also promote hyperglycemia and polyol-induced OxS.

As indicated in Figure 3, mitochondrial OxS induces insulin resistance leading to increased plasma glucose, increased plasma AGEs, and increased polyol-induced NADPH consumption, all of which contribute to systemic OxS and inflammation. Moreover, both in vitro and in vivo evidence indicate that plasma-protein AGEs can induce insulin resistance by repression of GLUT4 expression in skeletal muscle [32]. This AGE-induced insulin resistance is an example of an amplifying OxS feedback loop, i.e., plasma AGEs further increase insulin resistance by amplifying glycemia and OxS. OxS can also contribute to beta-cell dysfunction and reduced glucose-induced insulin secretion, thereby further increasing the risk of hyperglycemia and T2D progression [33]. Both AGE formation and systemic OxS give rise to an increased risk of both macrovascular and microvascular damage [34,35].

## 8. The OptRedox Strategy for Preventing or Slowing the Progression of T2D

Most publications on the roles of natural antioxidants in T2D progression tend to focus on individual antioxidant molecules such as vitamin E and their roles in blocking OxS. There are, however, benefits that arise from reframing this focus to also include OptRedox strategies for preventing or slowing the progression of T2D. An OptRedox approach is more nuanced by also embracing dynamic physiological processes such as glycemic control and physical activity. As outlined in Figure 3, processes that increase insulin resistance and glycemia can also promote damaging OxS and T2D progression. In contrast, processes that decrease insulin resistance and glycemia can be considered as promoting an OptRedox strategy. As will be detailed below, both glycemic control and exercise can be viewed as natural processes promoting an OptRedox status [13]. Exercise, despite causing a transient oxidative OxS, subsequently induces skeletal muscle antioxidant enzymes that are beneficial for preventing T2D progression [36,37]. 

### 8.1. Glycemic Control as a Natural OptRedox Process 

In those with T2D, glycemic control is clinically defined as maintaining an HbA1C level of less than 7.0%, or 6.5% for more strict control [38]. Both fasting and PPG impact HbA1C levels. Woerle et al. have found that lowering PPG is essential for optimizing glycemic control. In contrast to good glycemic control, poor glycemic control in subjects with T2D or prediabetes results in decreased plasma total antioxidant capacity and increased lipid peroxidation (a marker of OxS) [39]. Physical activity/exercise is well a recognized critical factor for promoting glycemic control in individuals with T2D or prediabetes [40,41].

### 8.2. Physical Activity/Exercise as an OptRedox Lifestyle Factor

As emphasized by the American Diabetes Association (ADA), physical activity in T2D and prediabetes improves glycemic control while also reducing cardiovascular risk and promoting weight loss [40]. Moreover, the ADA suggests that “regular exercise may prevent or delay T2D development”. Light exercise provides an excellent example of an OptRedox lifestyle factor with an untapped potential to reduce OxS and slow T2D progression and, could potentially be cost-effectively implemented in a school setting [42]. 

### 8.3. Light-Intensity Walking as an OptRedox Lifestyle Factor That Reduces Postprandial Glycemia (PPG)

A recent meta-analysis in predominantly adults with overweight or obesity showed that as little as two to five minutes of light-intensity walking significantly reduced PPG levels compared to prolonged sitting [43]. This meta-analysis also found that the light-exercise-induced lowering of PPG was more effective in overweight individuals compared to individuals with obesity, suggesting that obesity imposes additional metabolic dysfunction [43,44]. Given the acknowledged importance of early intervention, it is critically important that this research be repeated in a pediatric population, perhaps, in a school setting. The potential near-future availability of real-time noninvasive blood glucose sensors on consumer smartwatches will rapidly accelerate research efforts focused on PPG as well as fasting hyperglycemia [45,46]. Twenty-four-hour glycemic profiles have shown a significant reduction in PPG in T2D subjects performing 15 min of strolling during each postprandial period compared to sedentary T2D subjects [41,47]. 

Short-duration light-intensity walking can lower PPG simply by promoting skeletal muscle consumption of glucose. Exercise can, however, also increase skeletal muscle uptake of glucose by promoting GLUT4 translocation to the plasma membrane as well as GLUT4 expression [48]. Both low- and high-intensity exercise can equally and immediately induce GLUT4 expression in human skeletal muscle [49,50]. The signaling pathway for exercise-induced translocation of GLUT4 is distinct from that of insulin (see above) and likely involves AMP-activated protein kinase (AMPK) and increased sarcoplasmic calcium [48]. 

## 9. Exercise, Reactive Oxygen Species (ROS), and OptRedox Status

As reviewed by Kawamura [51] there is a “consensus that exercise increases the production of free radicals”. It is recognized, however, that exercise duration and intensity are key determinants of measurable OxS [51,52]. Moreover, transient exercise-induced OxS can evoke both beneficial and adverse physiological processes depending on the intensity/duration of the OxS and body conditioning [36]. High levels of exercise-induced OxS can result in adverse damage to skeletal muscle, particularly in untrained individuals [53]. It has long been recognized that physiological levels of ROS play essential roles in cell signaling pathways [54,55]. This raises the possibility that high levels of exogenous chemical antioxidant supplements could interfere with cell signaling pathways (see below) [56].

### 9.1. Exercise-Induced ROS Production Has a Biphasic Impact on Skeletal Muscle Force Production

Powers et al. [53] have reviewed the evidence showing that exercise-induced ROS production has a biphasic impact on skeletal muscle force production, i.e., low levels of ROS production increase contractile force, but high ROS levels decrease force production. There is, therefore, an optimal level of exercise-induced ROS production (and redox status) required for maximal skeletal muscle contraction force. The molecular mechanism(s) for this biphasic effect is/are not fully understood, but this illustrates the need to consider complex physiological processes in determining an OptRedox status [53,57]. 

The relationship between exercise intensity and ROS production has been and continues to be, the subject of intensive research [51,58]. During low and moderate exercise, the lactate released from muscle tissue into blood can be absorbed without build-up. During vigorous exercise, however, the released lactate is released faster than it can be absorbed, i.e., this is termed the lactate threshold. Quindry, et al. [52] have shown that ROS production in humans primarily results from exercise intensities above the lactate threshold. Subsequent research has shown that exercise duration can also impact the rate of ROS production in humans [51]. Although beyond the scope of this review, the overall consensus is that the ROS produced during vigorous exercise can have both positive and negative effects. In addition to the potentially positive effect ROS can have on skeletal muscle function, ROS can also increase the expression of some enzymatic antioxidants. As will be discussed below, this effect may be blunted by natural exogenous chemical antioxidants such as vitamin E. 

### 9.2. Exercise-Induced Oxidative Stress and Induction of Enzymatic Antioxidants

As mentioned above, exercise has long been associated with an increased activity level of some endogenous antioxidant enzyme, e.g., skeletal muscle GPX [59]. It has also been hypothesized that the ROS generated during exercise could be essential for this induction of antioxidant enzymes by modulating signal transduction pathways [37,59]. If this hypothesis is correct, it raises the concern that antioxidant supplements could interfere with this induction. The research of Ristow et al. [37] shows that supplementation for four weeks with ascorbate (1000 mg/day) plus vitamin E (400 IU/day) blocked the exercise-induced expression of GPX1 in skeletal muscle biopsies from healthy young men. These investigators also found that four weeks of exercise increased insulin sensitivity, but this effect was blocked by daily supplementation with ascorbate plus vitamin E. The form of vitamin E used in these experiments was RRR-alpha-tocopherol (more on this below). Ristow et al. concluded that exercise-induced ROS are essential for promoting insulin sensitivity and the induction of GPX1 [37]. Although discussed as being relevant to T2D, the subjects in this study were healthy adult males. In the context of T2D, the lifelong implementation of regular exercise and physical activity, in general, is overwhelmingly viewed as an essential factor for preventing or slowing T2D progression [60].

## 10. Sleep and Psychological Stress as Lifestyle Factors Affecting T2D and Oxidative Stress

In addition to exercise, dealing with other lifestyle factors that promote or cause sleep disorders/disturbances and/or psychological stress may also play important roles in T2D prevention and therapy [61,62]. Moreover, both sleep disorders [63,64] and psychological stress [65] have been linked to increased OxS. T2D is associated with a higher incidence of sleep disorders and emerging evidence supports a positive role of sleep quality in T2D glycemic control [61]. As detailed in Section 5 and Section 6, and Figure 3, poor glycemic control contributes to OxS. In an animal model, Everson et al. found that sleep deprivation was associated with a decreased level of liver catalase activity (an antioxidant enzyme) and GSH content (see Figure 2). Moreover, these authors found that sleep recovery could restore both liver catalase and GSH content to normal levels. The relationship between OxS and sleep disorders is attracting increased research interest [66]. Future research may help define the molecular mechanisms connecting sleep, antioxidants, and T2D. Due to preexisting OxS, individuals with T2D may have an enhance adverse susceptibility to any additional OxS driven by sleep disturbances and/or stress.

## 11. The Distinct Forms of Vitamin E and Their Effects on T2D Progression 

Natural chemical antioxidants that prevent or inhibit skeletal muscle mitochondrial oxidative stress would be excellent candidates for potentially slowing or blocking the progression of insulin resistance. Vitamin E is considered the primary lipid-soluble antioxidant and is present in all biomembranes including those of mitochondria where vitamin E levels can be increased by dietary supplementation [67].

### 11.1. Natural Vitamin E and the Importance of Stereochemistry

According to the International Union of Pure and Applied Chemistry the term “vitamin E” refers to “all tocol and tocotrienol derivatives exhibiting qualitatively the biological activity of α-tocopherol” (http://iupac.qmul.ac.uk/misc/toc.html, accessed on 3 May 2023). Natural vitamin E from dietary sources consists of (1) four tocopherol monophenol vitamers (TOH), i.e., alpha-TOH, beta-TOH, gamma-TOH, and delta-TOH) and; (2) four tocotrienol (T3) monophenol vitamers, i.e., alpha-T3OH, beta-T3OH, gamma-T3OH, and delta-T3OH. T3s are forms of vitamin E in which the hydrophobic “tails” have three trans double bonds [68].

The stereochemistry of natural vitamin E vitamers is very specific. For example, natural alpha-tocopherol has three asymmetric carbons, and each has the R stereochemical designation, i.e., 2*R*,4′*R*,8′*R*-alpha-tocopherol (or RRR-alpha-TOH). In contrast, synthetic alpha-tocopherol has both R and S configurations at each of the three asymmetric carbons and is, therefore, an equimolar mixture of eight compounds with only one eighth being the natural RRR-alpha-TOH: the other seven forms being xenobiotics with mostly unknown physiochemical properties. The synthetic form of alpha-TOH is most often termed all-racemic-alpha-tocopherol (or all-rac-alpha-TOH). Since vitamin E compounds have signal-transduction properties that are independent of their antioxidant oxidant abilities, it is important to detail the form used in any experiment/trial [69,70]. Unfortunately, this is often not the case, which makes reproducibility difficult, obscures potential physiological effects unique to specific vitamin E vitamers, and confounds meta-analyses which often lump all forms of vitamin E together. 

### 11.2. Supplementation with “Vitamin E” May Be a Valuable Strategy for Controlling Diabetes Complications

A meta-analysis of the effects of antioxidant vitamins on T2D concluded that supplementation with vitamin E “may be a valuable strategy for controlling diabetes complications” [71]. In this meta-analysis, the different forms of vitamin E were lumped together making it difficult to determine if positive or negative effects were unique to a form of vitamin E. In addition, the primary papers sometimes fail to distinguish all-rac-alpha-TOH from RRR-alpha-TOH. Nevertheless, all vitamin E vitamers are free radical quenchers and inhibit lipid peroxidation. It is possible, therefore, that the antioxidant properties shared by all members of the vitamin E family contribute to their positive effects on controlling T2D complications. Interestingly, increased lipid peroxidation is associated with poor glycemic control in T2D patients [39].

### 11.3. All-Racemic-Alpha-Tocopherol (All-Rac-Alpha-TOH) and Rice Bran Tocopherol Concentrate Inhibit Skeletal Muscle Generation of Hydrogen Peroxide

Given the central importance of mitochondrial oxidative stress in T2D (see Figure 3), vitamin E has been advanced as an antioxidant with great therapeutic potential for T2D treatment. As detailed above, skeletal mitochondrial hydrogen peroxide emission may be an initiating event for the development of insulin resistance. In a rat model, Chow et al. have quite remarkedly demonstrated that dietary supplementation with either all-rac-alpha-TOH or a rice bran tocopherol concentrate (8.6% RRR-alpha-TOH, 5.5% RRR-beta-TOH, and 15.4% gamma-T3OH) markedly decreases skeletal muscle hydrogen peroxide generation in a dose-dependent manner [72]. The mechanism for this important effect is not well understood, and has not been studied in human skeletal muscle, but this implies that early supplementation with vitamin E could slow the progression of T2D. Future mechanistic research on the role of vitamin E on mitochondrial hydrogen peroxide emission is a high priority.

### 11.4. Vitamin E and/or Ascorbate Supplementation Improves Glycemic Control in T2D

In contrast to the work of Ristow et al. [37] (see Section 9.2) in which the subjects were adult healthy males, El-Aal et al. [73] conducted a clinical trial looking at the effects of vitamin E and/or ascorbate on adult T2D males who were all under metformin treatment. The results clearly show beneficial effects on glycemic control in those supplementing (for 90 days) with vitamin E alone (400 mg twice daily), ascorbate (500 mg twice daily) alone, or the combination of both. Unfortunately, it is not clear if RRR-alpha-TOH or all-rac-alpha-TOH was the form of vitamin E used in this trial. Nevertheless, this study strongly suggests that future studies should look at the potential beneficial effects of vitamin E supplementation on pediatric subjects with prediabetes or with T2D. Subjects with T2D or prediabetes with antioxidant deficiencies due to enhanced OxS may respond differently to antioxidant supplementation (or exercise) than healthy subjects with adequate antioxidant levels. 

### 11.5. The Tocotrienol-Rich Fraction (TRF) from Palm Oil May Be Beneficial in Both Prediabetes and T2D and in Preventing Early Diabetic Retinopathy 

Evidence from a randomized double-blind placebo-controlled clinical trial (the Vafa study) in T2D subjects with poor glycemic control shows that the tocotrienol-rich fraction (TRF) from palm oil (200 mg daily) for eight weeks lowered fasting blood glucose levels by 15% and improved indices of OxS [74]. An earlier study (the Baliarsingh study) in adult T2D subjects did not show any benefit of tocotrienols (60 days, 3 mg/kg body weight, twice daily) on fasting blood glucose, but these subjects had well-medication-controlled blood glucose levels at the start of the trial [75]. This earlier study did, however, show a marked decrease in atherogenic low-density lipoprotein cholesterol levels in the subjects taking the tocotrienol supplement. In both the Baliarsingh et al. study and the Vafa et al. study, the palm oil TRF was predominantly alpha-T3OH, beta-T3OH, gamma-T3OH, and RRR-alpha-TOH with only a small content (about 4–8%) of delta-T3OH. The low content of delta-T3OH in the TRF is noteworthy since delta-T3OH may be uniquely important in preventing T2D progression, as detailed below.

A recent study by Ho, et al. [76] showed that TRF (200 mg twice daily for 12 months) was effective in preventing early diabetic retinopathy compared to a placebo. Diabetic retinopathy is a very common microvascular complication of T2D and makes a major contribution to blindness and vision loss in adults [76]. 

### 11.6. Delta-Tocotrienol Shows Promise in Treating Prediabetes

Treatment options for prediabetes are limited, especially in the pediatric population [77]. Encouragingly, a recent randomized controlled pilot study looked at the effects of T3 purified from annatto seeds: this preparation was primarily delta-T3OH (90%) and 10% gamma-T3OH [78]. The prediabetic adult study population was not being treated with any medications for glucose control or taking any dietary supplements. After 12 weeks of 300 mg/day of T3s, there were significant improvements in glycemic control compared to the controls taking a placebo [78]. T3s are thought to improve glycemic control by upregulating the skeletal muscle expression of GLUT4, insulin receptor substrate 1, AKT signaling, and by activation of peroxisome proliferator-activated receptor-gamma (PPAR-gamma) [78,79].

## 12. Vitamin C (L-Ascorbate or AA) and Its Role in T2D Progression and Management

**Figure 4:**
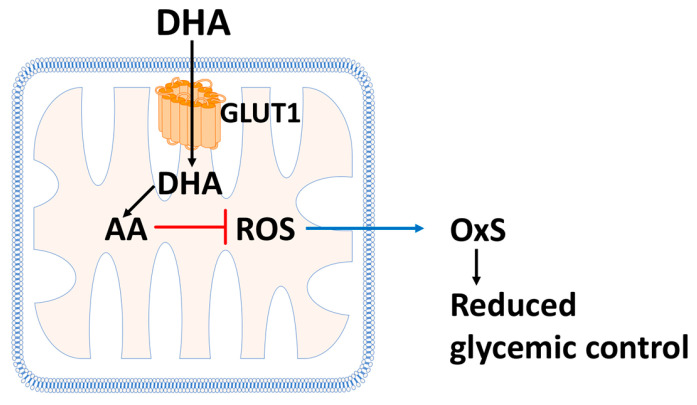
Vitamin C protects mitochondria from reactive oxygen species (ROS). Oxidized vitamin C (DHA, as shown in Figure 5) is transported from the cytoplasm into the mitochondrial matrix by GLUT1, where it is reduced to ascorbate (AA, as shown in Figure 5) where it blocks mitochondrial OxS by reducing the levels of ROS such as superoxide, hydrogen peroxide, and hydroxyl radicals. Reduced mitochondrial OxS may be an important mechanism for combating insulin resistance.

Vitamin C is a water-soluble antioxidant present in plasma and tissues. As detailed below, vitamin C status and vitamin C supplementation play potential roles in T2D progression and management [80,81,82,83,84,85,86,87]. The molecular mechanisms for the protective effects of vitamin C on T2D are not fully understood but inhibition of mitochondrial OxS is a probable candidate (see Figure 4). As discussed further below, Vitamin C may also reduce the chronic low-grade inflammation associated with promoting T2D progression [88]. 

### 12.1. Vitamin C Protects Mitochondria from Oxidative Stress

**Figure 5:**
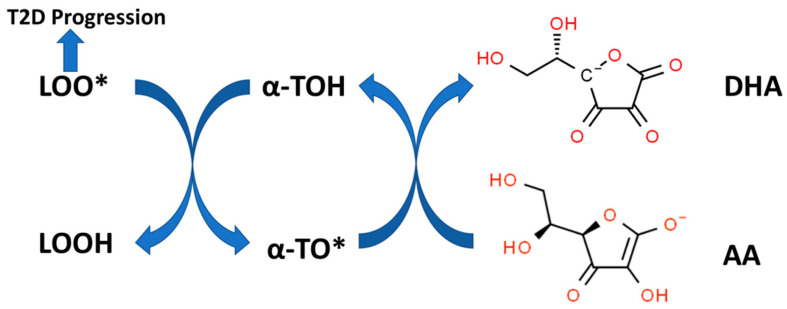
Vitamin E recycling by Vitamin C. Alpha-tocopherol (alpha-TOH) quenches lipid peroxidation by converting lipid peroxyl radicals (LOO*) to lipid hydroperoxides (LOOH) with the formation of alpha-tocopheroxyl radicals (alpha-TO*). The reduced form of vitamin C (AA), can recycle alpha-TO* back to alpha-TOH with the formation of oxidized vitamin C (DHA). Increased lipid peroxidation is associated with T2D progression (see text).

Figure 4 illustrates a possible mechanism whereby L-ascorbate (AA) supplementation could promote glycemic control. At pH 7.4, vitamin C is primarily present as the mono-anion as indicated in Figure 5 [89]. Outstanding in vitro work by Sagun et al. [90] has shown that DHA enters mitochondria, via the facilitative D-glucose transporter GLUT1, where it is reduced to AA and protects mitochondria from OxS damage. In oxidatively stressed purified mitochondria, AA was found to reduce the levels of H_2_O_2_, O_2_*^−^, and *OH (the hydroxyl radical) [90]. As discussed above (see Section 4), mitochondrial OxS is a key factor promoting reduced glycemic control and T2D progression (also see Figure 3). In vitro and ex vivo studies have demonstrated that mitochondrial OxS causes insulin resistance by impairing insulin-regulated GLUT4 translocation to the surface of myotubes (fused skeletal muscle cells) and adipocytes (see Section 3 and Section 4 above) [20]. 

### 12.2. Vitamin C and Oxidative Stress in T2D

Excess OxS in T2D would be expected to increase the metabolic consumption of AA, thereby lowering its plasma, tissue, and mitochondrial concentrations. Published data consistently shows lower plasma AA levels in patients with T2D (compared to healthy controls), but a lower dietary intake of AA might also be a contributing factor [87,91,92,93]. This issue has been addressed by both Bansal et al. [91] and Sinclair et al. [92] with the conclusion that the lower plasma levels of AA observed in patients with T2D are due to increased “demand” rather than inadequate dietary intake. Data provided by Bansal et al. indicates that, even with the daily recommended dietary intake (RDI) of AA, patients with T2D have about half the plasma AA levels found in healthy controls (consuming the RDI) [91]. 

An important caveat here is, however, the discovery that renal leakage can also contribute to low T2D plasma ascorbate levels and that poor glycemic control, microvascular complications, and obesity correlate with this renal leakage [83]. Chronic kidney disease is very common in patients with T2D and therefore urinary loss of AA can be of real clinical concern [94]. 

### 12.3. Vitamin C Supplementation Plays a Positive Role in Adult T2D Management and Progression 

An increasing body of literature suggests that AA supplementation plays a potential role in the management of T2D [80,81,82,83,84,85,86]. A 2021 meta-analysis by Mason et al. [80] shows that supplementation with AA decreases HbA1c, fasting glucose, and PPG. A noted limitation in this meta-analysis is, however, the lack of randomized controlled trials (RCTs) with sufficient power, as well as short-term study durations (less than six months) [85]. Moreover, the dose of AA used in intervention studies may be an important variable. An RCT in 2007 found that a daily 1000 mg dose, but not a 500 mg dose (for six weeks), was effective in reducing fasting blood glucose and HbA1c in adult patients with T2D [95]. 

Importantly, all the studies referenced in the above paragraph were conducted with adult populations. Unfortunately, very limited information is available on the potential role of AA in either the progression or management of children with T2D. An epidemiological study of 2025 UK children found that lower plasma AA concentrations were associated with insulin resistance and higher fasting glucose [96]. Future studies are urgently needed to test the hypothesis that increasing plasma AA could help prevent or slow T2D progression in children [96,97]. 

### 12.4. Do Synergistic Interactions between Vitamin C and Vitamin E Play a Role in Preventing T2D Pathophysiology?

Compelling in vitro evidence in model systems supports a synergism between alpha-TOH and AA in preventing lipid peroxidation [89,98,99,100]. The molecular basis for this in vitro synergy is outlined in Figure 5. AA, despite being water-soluble, can regenerate alpha-TOH (lipid-soluble) from the alpha-tocopheroxyl radical (alpha-TO*) present at the surface of biomembranes [101]. Alpha-TO* radicals are formed by quenching the lipid peroxyl radicals (LOO*) formed during lipid peroxidation with the concurrent formation of lipid hydroperoxides (LOOH). As shown in Figure 2, LOOH can be detoxified by the glutathione peroxidase system. LOO* radicals, if not quenched by alpha-TOH (or other forms of vitamin E), can promote rapid lipid peroxidation propagation reactions in biomembranes or other lipid–protein complexes (e.g., lipoproteins) with polyunsaturated fatty acid (PUFA)-containing lipids [102]. Lipid peroxidation and the chemical products of this process, such as malondialdehyde (MDA) and 4-hydroxy-2-nonenal (4-HNE), can cause functional damage to lipid–protein complexes and alter many physiological processes [102]. Biomarkers of lipid peroxidation in T2D patients have been associated with systemic inflammation [103], nephropathy, retinopathy [104], poor glycemic control, and T2D progression [39].

Sato et al. [105] recently presented data from an animal model showing that vitamin C can spare alpha-tocopherol (stereochemistry not specified) in liver and heart tissue but not in other organs that were examined (or plasma). These data show that any sparing in vivo effect of ascorbate on alpha-TOH levels is likely to be tissue/organ-specific. The potential physiological significance of any in vivo vitamin E/C synergy has yet to be firmly established. Most in vivo evidence suggests that such a synergism may be relatively unimportant [98]. A long-term RCT study (12 months) in healthy men found that vitamin E alone (182 mg/day of RRR-alpha-TOH) or in combination with slow-release vitamin C (500 mg/day) reduced in vivo serum-lipid-peroxidation markers (compared to placebo) but vitamin C alone did not [106]. A subsequent in vivo RCT study by Huang et al. [107] in healthy adults looked at daily supplementation (for two months) with vitamin C (500 mg), vitamin E alone (400 IU of RRR-alpha-tocopheryl acetate), or the combination of both. This study found that supplementation with vitamin C alone or vitamin E alone reduced in vivo lipid peroxidation but there was no synergistic effect of supplementing with both vitamins.

It should be kept in mind that most studies attempting to look at the potential in vivo vitamin E/C synergism focus on systemic markers of lipid peroxidation in plasma/serum or urine and not at OxS markers in subcellular organelles, e.g., skeletal muscle mitochondria. In vivo vitamin E/C antioxidant synergy (short and/or long-term) could potentially be important in improving T2D pathogenesis biomarkers other than fasting serum/plasma systemic OxS markers. This possibility has not been adequately tested. Encouragingly, a small RCT study with vitamin C supplementation (1000 mg/day for six weeks) alone found that postprandial OxS (see Section 6) was reduced compared to placebo [108]. Neri et al. found that a daily antioxidant supplement mixture with N-acetylcysteine (600 mg), alpha-TOH (300 mg), and vitamin C (250 mg) reduced postprandial OxS after a moderate-fat meal in healthy adults and patients with T2D: individual antioxidants were not studied [109]. 

Both in vitro and in vivo studies tend to focus on vitamin E in the form RRR-alpha-TOH and not the tocotrienol forms, which (see Section 11.5 and Section 11.6) may be particularly important in T2D pathophysiology. Moreover, plasma/tissue levels of tocotrienols are much lower than those of tocopherols after supplementation and therefore may be more sensitive to OxS depletion and any potential for tocotrienol/vitamin C synergy.

## 13. Dietary and Supplemental Manganese and T2D

Manganese (Mn) is an essential trace element and a cofactor for the antioxidant enzyme Mn-superoxide dismutase (MnSOD) which is the sole isoform of SOD located in the mitochondrial matrix (see Figure 2). Since mitochondria are a major subcellular site of ROS production, MnSOD is thought to play an important role in preventing ROS-induced cellular damage [110]. As covered above in Section 4, this strongly suggests that MnSOD could play a role in preventing or reversing T2D progression.

The potential roles of Mn status, MnSOD levels, and OxS in T2D have been the subject of both animal and human studies [111,112,113,114,115]. These studies reveal a complex pattern that will require further research. In wild-type mice, Lee et al. [111] have demonstrated that Mn supplementation (by injection for 8 weeks) resulted in increased liver MnSOD activity (by 73%) and protection against high-fat-diet-induced diabetes. These authors provide evidence showing that the Mn injection increased insulin secretion and suggest that this effect, along with reduced mitochondrial OxS, could explain the in vivo protection against high-fat-diet-induced diabetes.

Utilizing Zucker diabetic fatty (ZDF) rats as a T2D model, Burlet et al. [112] explored the hypothesis that supplemental Mn (by gavage for 7 weeks) could help prevent vascular inflammation and endothelial dysfunction. In this ZDF model, Mn supplementation reduced glucose-induced monocyte adhesion to endothelial cells as well as endothelial dysfunction. Complementary in vitro experiments showed, however, that this effect was not dependent upon MnSOD expression [112]. This unexpected finding does not rule out other beneficial roles of MnSOD in T2D but does suggest that supplemental Mn has pleiotropic physiological effects. 

Although very informative, the role of Mn in human T2D progression has primarily been limited to association/prospective-cohort studies rather than RCTs. Wang et al. [114] looked at the association between serum Mn levels and prediabetes in Chinese adults. This study found an inverse association between serum Mn levels and prediabetes suggesting that some populations (particularly women) could potentially benefit from a Mn-rich diet [114].

In prospective cohort studies, Du et al. [113] found that the incidence of T2D was inversely associated with dietary Mn levels. These investigators also utilized a food-frequency questionnaire to calculate the total dietary antioxidant capacity (TAC) for each subject. Dietary TAC provides an overall measure of the total antioxidant capacity arising from complex mixtures of dietary antioxidants (e.g., polyphenols and flavonoids) [116]. As reported in the Rotterdam study, dietary TAC is related to a lower risk of T2D and is inversely associated with insulin resistance [116]. Du et al. [113] found that the inverse association of dietary Mn with T2D incidence was independent of TAC suggesting that a high TAC does not “mask” the beneficial effect of dietary Mn. A recent cross-sectional study by Chen et al. [115] looked at the association between serum Mn levels and T2D in a Chinese population with hypertension. These authors found a U-shaped association between serum Mn levels and the T2D odds ratio suggesting that there could be an optimal moderate level of serum Mn for prevention of T2D [115].

The human and animal Mn data reviewed here looks promising for preventing or treating T2D. A strong molecular argument can be made that dietary/supplemental Mn reduces mitochondrial OxS. Nevertheless, there is a notable lack of supporting RCTs. Hopefully, this gap will be the focus of future research.

## 14. Selenium Supplementation and T2D

Selenium (Se) is an essential trace element and is required for the activity of GPX1–4 and 6, with GPX1 being the most abundant form of the GPXs [117]. Rats deficient in Se exhibit in vivo evidence of OxS, particularly in the retina [118]. A small longitudinal study in 2019 suggests that Se supplementation (200 microgram/daily for six months) in patients with T2D showed improvement in glycemic control and decreased low-density lipoprotein cholesterol levels. A comprehensive review of the role of Se in T2D by Wong et al. [119] provides, however, a critical caution on the blanket use of supplemental Se for individuals with T2D who already have a diet with adequate Se intake. The basis for this caution lies in the U-shaped curve for the dose of Se versus health benefit [119]. This caution should be emphasized for pediatric patients where evidence for the potential role of Se supplementation in T2D progression is minimal.

## 15. The Role of Beta-Carotene in T2D Prevention

Beta-carotene is the most abundant carotenoid in humans and food and has long been recognized for its antioxidant and anti-inflammatory properties [120]. A comprehensive review suggests that beta-carotene, either in food or as a dietary supplement, may have “a vital influence” in the prevention of T2D [120]. A 2021 meta-analysis of prospective observational studies strongly supports the role of dietary and circulating carotenoids in reducing the risk of T2D [121]. Nevertheless, a very large and long-term RCT indicates that beta-carotene supplementation (50 mg on alternate days for 12 years) in healthy men did not affect the risk of subsequent T2D. While a diet rich in carotenoids looks very promising for reducing T2D risk, the lack of confirming RCT studies with a beta-carotene supplement remains unresolved [121]. Foodstuffs rich in beta-carotene may likely contain other complex mixtures of bioactive nutrients that act synergistically to reduce T2D risk. 

## 16. Conclusions

The evidence reviewed here strongly supports the role of OxS and ROS in both the initiation and progression of T2D. The role of natural antioxidant products in combating T2D is best evaluated in the context of the complex physiological processes associated with its initiation and progression. The consumption of high-calorie, high-fat diets gives rise to transient insulin resistance resulting from mitochondrial OxS. In susceptible individuals, gradual increases in PPG and fasting blood glucose promote systemic OxS with amplifying feedback loops that eventually can cause OxS-induced beta-cell dysfunction and T2D with micro-and macro-vascular damage (see Figure 3). Avoiding high-calorie, high-fat diets, engaging in exercise, and avoiding sleep disturbances are, in effect, key frontline antioxidant strategies. The role of natural antioxidant compounds in combating T2D must likewise be considered beyond their simple roles as inhibitors of in vitro or ex vivo OxS. Vitamin E illustrates this point. While all members of the vitamin E family have powerful in vitro antioxidant properties, they can also exhibit distinct abilities to regulate cellular signal transduction pathways important to T2D progression. While most literature on the role of vitamin E vitamers in T2D or prediabetes has been limited to RRR-alpha-TOH or all-rac-alpha-TOH, emerging trials with tocotrienols show promise. As detailed in Section 11.6, a delta-T3-rich supplement improved glycemic control in adults with prediabetes. 

The evidence reviewed here supports the very positive role of vitamin C supplementation in T2D prevention and management. Moreover, decades of research attest to the nontoxicity of vitamin C supplementation. In contrast, both Mn and Se supplementation data exhibit U-shaped risk versus dose–response curves, suggesting the importance of careful quality control issues and the need to establish clinical criteria for safe dosing. For beta-carotene, the lack of confirming RCT data limits any suggestion for its use as a supplementation in T2D prevention/management.

An additional way of promoting the OptRedox strategy in clinical settings is utilization of the Bright Futures guidelines which recommend educating patients and parents on the importance of a healthy diet and exercise beginning at age two [122]. Healthcare providers should assess dietary and physical activity behaviors and make age-appropriate recommendations. Despite these recommendations, childhood obesity rates continue to rise, which contributes to the rise in T2D. An emphasis on prevention strategies such as a healthy diet and exercise must be accompanied by a better understanding of human motivation. 

High-intensity interval training is recognized as an effective means of promoting a healthy body composition, OptRedox, and for reducing T2D risk. Nevertheless, the optimal exercise duration and rest intervals for children and adolescents remain unclear [123]. The role of sleep disorders in promoting both T2D risk and OxS suggests that better preventative strategies are needed to address these issues. Lifestyle modifications, while proven to be effective, are some of the hardest for patients and families to achieve.

Although there is a consensus that early diet, exercise, and effective nutraceutical interventions are optimal for preventing or reversing T2D progression, most research has focused on adults. It is critical, therefore, that more research be promoted with pediatric populations.
